# Analysis of MicroRNA Expression Profiling Involved in MC-LR-Induced Cytotoxicity by High-Throughput Sequencing

**DOI:** 10.3390/toxins9010023

**Published:** 2017-01-07

**Authors:** Junguo Ma, Yuanyuan Li, Lan Yao, Xiaoyu Li

**Affiliations:** College of Life Science, Henan Normal University, Xinxiang 453007, Henan, China; mjunguo_1378@126.com (J.M.); 15993030397@126.com (Y.L.); yl529400872@126.com (L.Y.)

**Keywords:** MC-LR, HepG2, microRNA, high-throughput sequencing, cytotoxicity

## Abstract

In recent years, microRNAs (miRNAs) in toxicology have attracted great attention. However, the underlying mechanism of miRNAs in the cytotoxicity of microcystin-LR (MC-LR) is lacking. The objective of this study is to analyze miRNA profiling in HepG2 cells after 24 h of MC-LR-exposure to affirm whether and how miRNAs were involved in the cytotoxicity of MC-LR. The results showed that totally 21 and 37 miRNAs were found to be significantly altered in the MC-LR treated cells at concentrations of 10 and 50 μM, respectively, when compared to the control cells. In these two groups, 37,566 and 39,174 target genes were predicted, respectively. The further analysis showed that MC-LR-exposure promoted the expressions of has-miR-149-3p, has-miR-449c-5p, and has-miR-454-3p while suppressed the expressions of has-miR-4286, has-miR-500a-3p, has-miR-500a-5p, and has-miR-500b-5p in MC-LR-treated groups when compared to the control group. Moreover, the result of qPCR confirmed the above result, suggesting that these miRNAs may be involved in MC-LR-hepatotoxicity and they may play an important role in the hepatitis and liver cancer caused by MC-LR. The target genes for differentially expressed miRNAs in MC-LR treatment groups were significantly enriched to totally 23 classes of GO, in which three were significantly enriched in both 10 and 50 μM MC-LR groups. Moreover, the results of KEGG pathway analysis showed that MC-LR-exposure altered some important signaling pathways such as MAPK, biosynthesis of secondary metabolites, and pyrimidine and purine metabolism, which were possibly negatively regulated by the corresponding miRNAs and might play important role in MC-LR-mediated cytotoxicity in HepG2 cells.

## 1. Introduction

Microcystins (MCs) are potent hepatotoxins and secondary metabolites produced by some cyanobacteria, *Microcystis*, *Oscillatoria*, *Aphanizomenon*, *Planktothrix*, *Nostoc*, or *Anabaena*, occurring worldwide in freshwater [1]. MCs are highly toxic to aquatic organism, wildlife, livestock, and human, and the outbreaks of poisoning effects on human health caused by MCs have been reported in many countries [2,3,4,5]. The acute intoxication of MCs can lead to human death [6] while chronic exposure to MCs has been verified to be one of the risk factors for the high incidence of human primary hepatocellular cancer [7,8]. In toxicology, the toxicity mechanism of MCs is primarily due to its intense inhibition of intracellular protein phosphatases 1 (PP1) and phosphatases 2A (PP2A), which causes hyperphosphorylation of key functional proteins and induces cellular metabolic disturbance and cytoskeleton damage [9,10]. Meanwhile, MCs can induce excessive reactive oxygen species (ROS) in cells that consequently leads to oxidative stress and promotes lipid peroxidation in the cells, which is considered as another important mechanism of MCs hapetotoxicity [11,12,13]. However, the detail molecular mechanism of MCs-toxicity in hepatocytes remains poorly clear due to a multi-pathway process of MCs-toxicity.

MicroRNAs (miRNAs) are small single-stranded non-coding RNAs with an average of 22 nucleotides and they can negatively regulate the expressions of their target genes and control many developmental and cellular processes in eukaryotic organisms by binding to recognition sequences usually on 3′-untranslated regions of mRNAs, resulting in miRNA-mediated degradation of target gene and translational repression or decay [14,15,16]. It is reported that more than 60% of human protein-coding genes contain miRNA binding sites and may be regulated by miRNAs [17]. Although the physiological function of the most of miRNAs is still largely unknown, increasing experimental evidences strongly indicate that miRNAs are involved in controlling a wide range of biological processes [18,19]. They likely play important roles in a number of human diseases, for example liver disease and cancer [20] and they may be promising biomarkers of diseases. Nowadays, there have been quite a few reports indicating that miRNAs have attracted great interest in toxicological study [21,22] and they may play important role in cellular responses to xenobiotic stress [23].

Recently, published work via quantitative real-time PCR (qPCR) and miRNA microarray have provided convincing evidences that miRNAs are probably involved in the MCs-toxicity and hepatitis and tumorigenicity induced by MCs [24,25]. Brzuzan et al. [26,27] found that let-7c and miR-122 were significantly up-regulated in whitefish liver after MCs-exposure and they supposed that miR-122-5p might be a plasma biomarker of liver damage in whitefish caused by microcystin-LR (MC-LR) [28], which is found to be the most common and potent variant in MCs [29]. Meanwhile, a similar result was obtained in mouse liver according to report by Zhao et al. [25]. Recently, a report based on miRNA-Seq transcriptome profiling analysis showed that totally 73 and 83 miRNAs were found to be significantly differentially expressed in the liver of juvenile whitefish exposed to 100 μg·kg^−1^ of MC-LR for 14 and 28 days, respectively [30]. Moreover, Zhou et al. [31,32] found that miRNAs were involved in the reproductive toxicity of male mice caused by MC-LR in which miR-98-5p, miR-758, and miR-541 might play important role [33,34]. In general, most miRNA expressions altered in animal tissues or in vitro cells after MCs-exposure [35]. Therefore, it is reasonable to assume that miRNAs are involved in MCs cytotoxicity and they might play roles in human hepatitis and primary liver cancer caused by MCs.

High-throughput sequencing is a very useful and high-efficiency method, which can provide an insight into identification of miRNA function. The present study aimed to determine microRNA expression profiling in the cytotoxicity of MC-LR on the human hepatocellular carcinoma (HepG2) cells by using miRNA high-throughput sequencing in order to elucidate the toxicity mechanism of MCs. Results of this study would represent a novel mode of action of MC-LR exposure on HepG2 cells and provide insight into the mechanism of MCs hepatotoxicity in human. Meanwhile, the specific alteration in miRNA expression may be a promising biomarker for MCs-induced toxicity on humans.

## 2. Result

### 2.1. Sequencing Summary of Small RNAs

In order to determine whether miRNAs differentially expressed in HepG2 cells treated by MC-LR, small RNA (sRNA) libraries were constructed and submitted to Illumina/solexa sequencing. After deep sequencing, 11,883,540, 12,304,052, and 11,856,736 raw reads were obtained from the sRNA library of control, 10 μM, and 50 μM MC-LR-treated HepG2 cells, respectively. After data cleaning, 11,588,775, 12,003,916, and 11,387,206 clean reads were generated from the three sRNA libraries, accounting for 98.07%, 98.13%, and 96.49% of total reads of the control and two treatment groups, respectively (Table S1). Then, sRNAs ranging between 18 nt and 30 nt in length were subjected to further analysis. The distributions of selected reads were analyzed, as shown in Figures S1 and S2. The majority of reads accounting for above 90% in the sRNA libraries were in range of 20 to 24 nt in length, most of which contain 5′ A or 5′ U. Tags align to Genbank database and Rfam database with blast, screen and remove rRNA, scRNA, snoRNA, snRNA, and tRNA associated tags, and tags map to exon and intron with processes, screen and remove exon and intron associated tags, and then summary of each RNA alignment are shown in Figure S3. The total amount rRNA in control, 10 μM, and 50 μM MC-LR-treated cells was 1.92%, 2.11%, and 2.26%, respectively.

### 2.2. miRNA Expression Profile in MC-LR Treated HepG2 Cells

Referenced to miRBase, expressions of 851, 815, and 833 known human miRNAs were detected in HepG2 cells from the control, 10 μM, and 50 μM MC-LR treatment groups, respectively (Table S2). A comparison of miRNA expressional levels between control and MC-LR-treated HepG2 cells revealed that totally 21 miRNAs were significantly altered in HepG2 cells in 10 μM MC-LR group, of which five were significantly up-regulated while 16 were down-regulated (Table 1). Meanwhile, in 50 μM group, 37 miRNAs were significantly altered (20 up-regulated and 17 down-regulated) (Table 2). Particularly, among the varied miRNAs, expressions of has-miR-149-3p, has-miR-449c-5p, and has-miR-454-3p were evidently promoted while has-miR-4286, has-miR-500a-3p, has-miR-500a-5p, and has-miR-500b-5p were significantly down-regulated in both 10 and 50 μM MC-LR groups.

### 2.3. Differentially Expressed miRNAs Validated by qPCR

**Figure 1:**
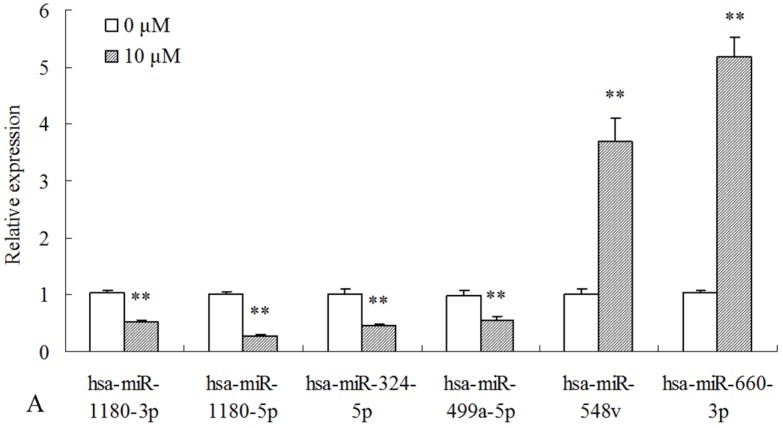

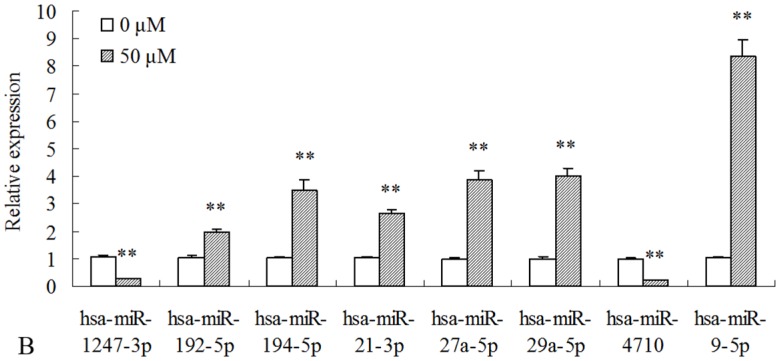
Transcriptional levels of miRNAs in the HepG2 cell after 24 h of 10 μM or 50 μM MC-LR exposure: (**A**) 10 μM MC-LR; and (**B**) 50 μM MC-LR. MC-LR-exposure and miRNA level determination in HepG2 cells are described in the Section 4.2 and Section 4.8. Asterisks denote a response that is significantly different from the control (* *p* < 0.05, ** *p* < 0.01).

To validate the miRNA expression profiles, we determined the expressional levels of 14 miRNAs by qPCR. According to the analysis results of miRNA expression profiles, two miRNAs (has-miR-548v and has-miR-660-3p) were up-regulated, while four miRNAs (has-miR-1180-3p, has-miR-1180-5p, has-miR-324-5p, and has-miR-499a-5p) were down-regulated in 10 μM MC-LR-exposure groups in comparison with control group (Figure 1A). Meanwhile, has-miR-1247-3p and has-miR-4710 transcription were suppressed in higher concentration group (50 μM), while has-miR-192-5p, has-miR-194-5p, has-miR-21-3p, has-miR-27a-5p, has-miR-29a-5p, and has-miR-9-5p were promoted (Figure 1B). The expression data obtained by qPCR analysis are comparable with the miRNA expression profiles determined by high-throughput sequencing.

**Figure 2:**
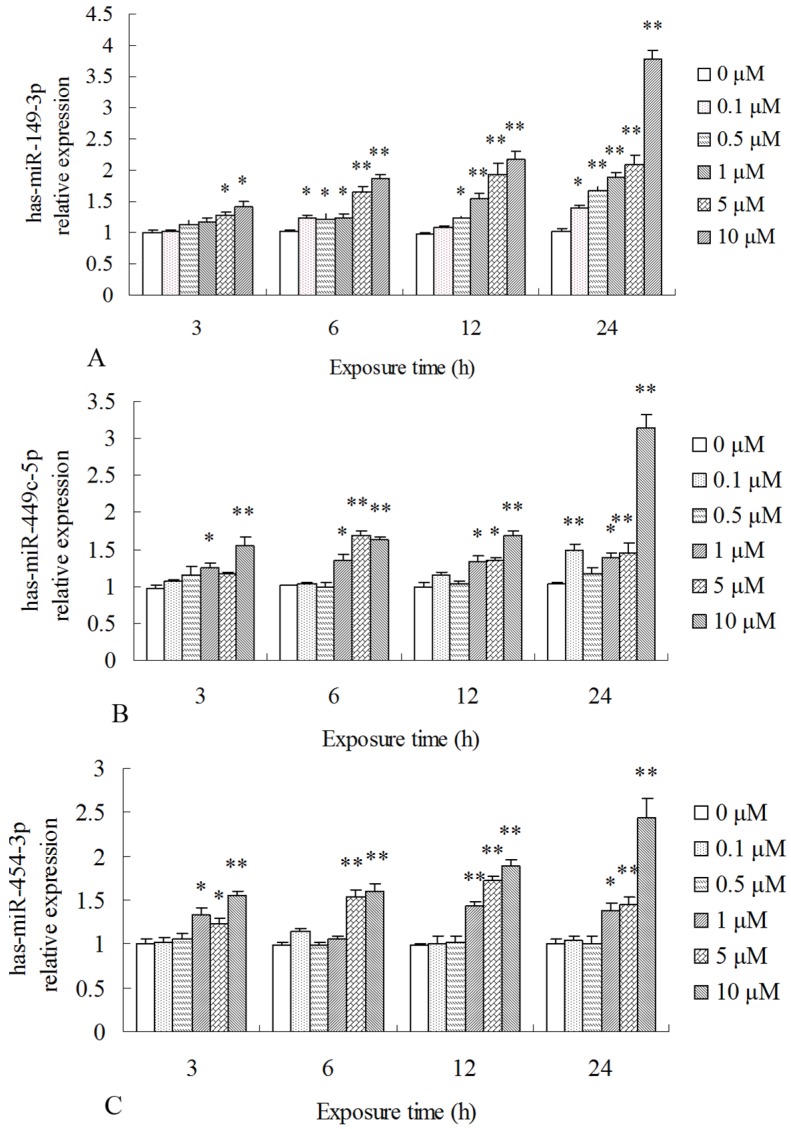

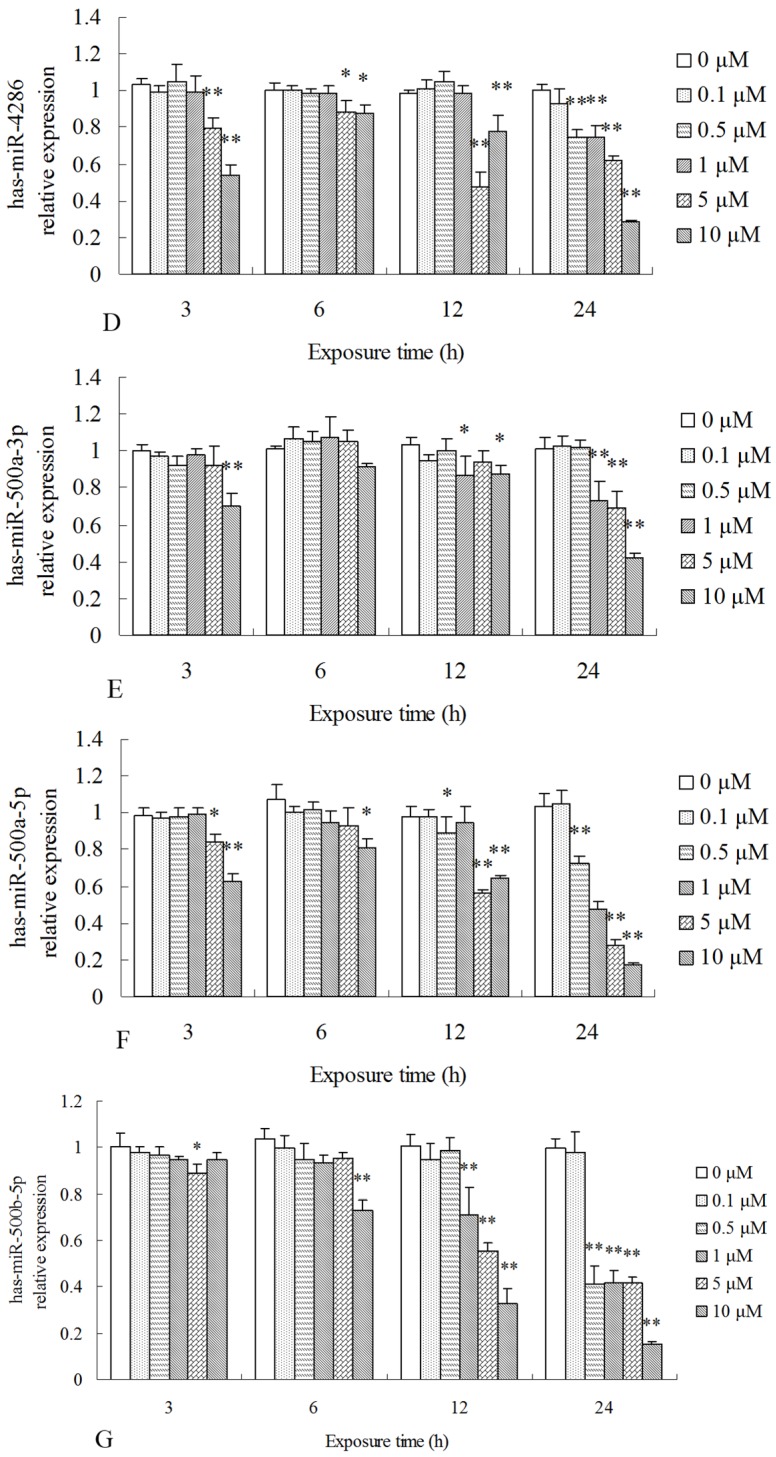
Transcriptional levels of the selected target miRNAs in the HepG2 cell after 24 h of MC-LR exposure: (**A**) has-miR-149-3p; (**B**) has-miR-4286; (**C**) has-miR-449 c-5p; (**D**) has-miR-454-3p; (**E**) has-miR-500 a-3p; (**F**) has-miR-500 a-5p; and (**G**) has-miR-500 b-5p. MC-LR-exposure and the miRNA level determination in HepG2 cells are described in the Section 4.2 and Section 4.9. Asterisks denote a response that is significantly different from the control (* *p* < 0.05, ** *p* < 0.01).

To further confirm if miRNAs were involved in MC-LR-induced cytotoxicity in HepG2 cells, we performed another cytotoxicity test and then detected the expressions of target miRNAs using qPCR. In the cytotoxicity test, HepG2 cells were exposed to 0.1, 0.5, 1, 5, and 10 μM MC-LR for 3, 6, 12, and 24 h, and RNA isolation and qPCR were performed as described above. The results of qPCR showed that MC-LR exposure significantly promoted the transcription levels of has-miR-149-3p, has-miR-449c-5p, and has-miR-454-3p while down-regulated has-miR-4286, has-miR-500a-3p, has-miR-500a-5p, and has-miR-500b-5p when compared to the control cells in a time- and dose-dependent pattern (Figure 2).

### 2.4. Putative Target Genes of the Differentially Expressed miRNAs and the Results of GO and KEGG Pathway Analysis

Differentially expressed miRNAs were subsequently taken to predict putative target genes by using targetscan and miRanda algorithm. The results showed that there were 37,566 and 39,174 putative target genes in 10 and 50 μM groups, respectively (Table S3). GO enrichment analysis showed that target genes related to 23 and three items were significantly enriched in 10 μM and 50 μM MC-LR-treated cells in comparison to genes in control cells, respectively (Tables S4 and S5). Furthermore, these predicted target genes classified according to KEGG pathway annotation revealed that they were significantly enriched in MC-LR-treated cells and mainly related to transcriptional misregulation in cancer, MAPK signaling pathway, purine and pyrimidine metabolism, and so on, suggesting that these signaling pathways may play important roles in MC-LR cytotoxicity (Tables S6 and S7).

### 2.5. Novel miRNAs Predicted

A total of 110 novel miRNAs were identified in all cells including control, 10 μM, and 50 μM groups, in which 18 same novel miRNAs were found. However, all of these novel miRNAs were in low expressional level (less than 300 reads) except for novel-mir-39 (above 18,000 reads) and novel-mir-14 (more than 300 reads) (Table S8 and Figure S4).

## 3. Discussion

miRNAs are non-coding RNAs that regulate gene expression at the post-transcriptional level by binding to particular mRNA target(s), which plays important role in a wide range of biological and pathological processes, such as developmental timing, signal transduction, cell proliferation, differentiation, neuronal disease, cancer, apoptosis, and metabolism [15,18,36,37]. Accumulating evidences suggest that miRNAs are not only involved in a lot of chronic disease and tumor induced by environmental toxicants, but also play important role in regulation the toxicity and detoxification of toxicants [38,39]. It is well known that MCs are widely distributed in fresh water during cyanobacterial blooms and they are highly toxic to aquatic organisms and even humans [40]. Hence, it is reasonable to believe that miRNAs are likely involved in the hepatitis or hepatocellular cancer associated with MCs exposure. In order to investigate the possible role of miRNAs in MCs toxicity, we conducted the high-throughput sequencing to determine the expression profiles of miRNAs in HepG2 cells after 24 h of MC-LR-exposure as to affirm whether and how miRNAs were involved in the cytotoxicity of MC-LR. Our results showed that totally 21 miRNAs were found to be significantly altered (five up-regulated and 16 down-regulated) in 10 μM MC-LR group when compared to the control. However, in 50 μM group, totally 37 miRNAs displayed altered expressions (20 up-regulated and 17 down-regulated). The result of qPCR confirmed the above result, suggesting that these miRNAs may be involved in MC-LR-hepatotoxicity. Moreover, we also found that MC-LR-exposure promoted the expressions of has-miR-149-3p, has-miR-449c-5p, and has-miR-454-3p while suppressed the expressions of has-miR-4286, has-miR-500a-3p, has-miR-500a-5p, and has-miR-500b-5p in both 10 μM and 50 μM groups when compared to the control group, suggesting that these miRNAs may play a role in MC-LR-associated hepatitis and liver cancer. Zhao et al. found that the expressions of 37 miRNAs were altered in the liver of mice after MC-LR exposure [25]. In another study, 31 miRNAs were found to be significantly affected at 72 h post-fertilization of zebrafish embryos induced by MC-RR [41]. Brzuzan et al. also found that MC-LR exposure changed the expressions of liver miRNAs in whitefish [27]. Our previous study also showed that MCs elevated the transcription levels of dre-miR-21 and dre-miR-27b while down-regulated the expressions of dre-miR-122 and dre-miR-148 in the liver of zebrafish intraperitoneally injected with MCs [42]. In another study, 126 miRNAs (78 up-regulated and 48 down-regulated) were found to be altered in MC-LR-treated WRL-68 cells compared to the untreated cells [24]. Importantly, several of these changed miRNAs (for example, miR-21, miR-122, and miR-221) were found to play important roles in maintaining normal physiological functions of these cells.

To identify the possible gene and pathway targeted by the aberrant miRNAs, we predicted the target genes ascertained from the GO database and KEGG pathway analysis [43]. A total of 37,566 and 39,174 target gene transcripts were identified from the differently expressed miRNAs in 10 and 50 μM MC-LR-treated cells, respectively. Three GO items were significantly enriched in 10 and 50 μM MC-LR-treated cells in comparison to control cells. Moreover, the results of KEGG pathway analysis showed that MC-LR-involved signaling pathways such as mitogen-activated protein kinase (MAPK), biosynthesis of secondary metabolites, pyrimidine metabolism, and purine metabolism were possibly negatively regulated by miRNAs, which might play an important role in the toxicity process of MC-LR in HepG2 cells. Early in 1999, Toivola and Eriksson hypothesized that MC might promote MAPK signaling pathways and MAPK be a key pathway for MCs-toxicity [44]. In recent years, more and more studies confirmed that MC-hepatotoxicity was mediated by MAPK pathway due to PP2A inhibition by MC [45,46,47,48]. In the present study, considerable target genes of aberrant miRNAs induce by MC-LR were also enriched to MAPK pathway, suggesting that the pathway may play a key role in MC-LR cytotoxicity on HepG2 cells via the negative regulation of miRNAs.

Mirdeep software was utilized to predict novel miRNAs by exploring the secondary structure, the Dicer cleavage site, and the minimum free energy of the unannotated small RNA tags which could be mapped to antisense exon, intron, and intergenic region of human genome. Our results showed that 110 novel miRNAs were identified in the three samples, in which there were 18 of the same novel miRNAs. Regrettably, there was no expressional change of these miRNAs in the MC-LR-treated cells compared to the untreated cells, suggesting that these miRNAs may be not directly responsive to MC-LR-exposure in HepG2 cells. However, the predicted and screened candidates of miRNAs will be useful for further enriching the resource of human miRNA database and may provide the basis for the future functional study.

It is well known that the MCs concentrations in the bloom-forming lakes or reservoirs are very low, usually around ng-μg·L^−1^ even during heavy cyanobacterial blooms [49]. However, it is worth noting that the exposure dose or concentration of MCs used for in vitro study on laboratory conditions was usually more than 1000-fold higher than the guideline value (approximately 1 nM) recommended by WHO [12,50,51]. Furthermore, quite a number of researchers found that in vitro models including both normal cells (HL7702) and cancer cells (HepG2) were usually insensitive to MCs-toxicity [12,51,52], which may be due to their different transport capacity or drug resistance to MCs. Previous researches have found that MCs is usually transported into cells via Oatps, for example, OATP1B1 and OATP1B3 are the most efficient MC-LR transporters in human hepatocytes [53,54]. An increasing number of reports have demonstrated that OATP transport system is preserved in HepG2 cells [55,56]. Moreover, our previous work also revealed that HepG2 cells could express OATP1B1 and OATP1B3 and MC-LR could enter HepG2 cells even if MC-LR concentration was as low as 0.1 nM. However, the low concentration of MC-LR (e.g., 0.5 or 5 µM) did not point out any elevated ROS level, and the generation of ROS was related to the toxicity of 10 and 50 µM MC-LR-treated HepG2 cells [12]. This result may be because of the fact that the low concentrations of MC-LR is not enough to induce the generation of ROS in HepG2 cells [51] or the excess ROS caused by MC-LR was captured and eliminated by the antioxidant enzymes, such as SOD, CAT, and GST, and nonspecific antioxidants, such as glutathione and vitamin E [57,58]. Žegura et al. demonstrated that 1 µM MC-LR could induce a time-dependent decrease of GSH levels in HepG2 cells [50]. Additionally, HepG2 cells can express antioxidant enzymes such as SOD, CAT, and GST on a par with or more than that of primary human hepatocytes [59], which might be another possible reason for the above result.

In summary, this work showed that in vitro administration of MC-LR affected miRNA expression profiles, and miRNA might play an important negatively regulated role in the cytotoxicity of MC-LR and the hepatitis or liver cancer induced by MC-LR. The current studies of miRNAs in MCs-related toxicity are often limited to miRNA profiling and identification, the precise mechanism remain uncharacterized although a few studies have investigated down-stream target genes [31,60]. Thus, well-designed follow-up studies to elucidate biological significance of altered miRNAs in MC-LR toxicity will be important in the future.

## 4. Materials and Methods

### 4.1. MC-LR

MC-LR (purity ≥ 95%) was obtained from the Express Technology Co., Ltd. (Beijing, China) and dissolved in sterilized double distilled H_2_O to a stock solution (1 mM) in a 1.5 mL centrifuge tube at −20 °C within one month. Before the experiment, the stock solution was thawed and then diluted to obtain the experimental concentrations with phosphate-buffered saline (PBS).

### 4.2. Cell Culture and MC-LR Exposure

HepG2 cells were obtained from the Henan Key Laboratory for Heredity Diseases and Molecular Targeted Medicines in Xinxiang, Henan, China and cultured in a 25 cm^2^-cell culture flask (1.5 × 10^6^ cells) with 5 mL RPMI-1640 medium (Sorlabo, Beijing, China) containing with 10% (*v*/*v*) heat-inactivated fetal bovine serum (FBS) (Sijiqing, Zhejiang, China), 100 U·mL^−1^ of penicillin, and 100 μg·mL^−1^ of streptomycin in a 5% CO_2_ humidified incubator at 37 °C. The logarithm growth cells were trypsinized by 0.25% of trypsin, collected, and then reseeded in a new 25 cm^2^-cell culture flask (1.5 × 10^6^ cells) every four day. Cells were trypsinized and reseeded for at most 20 times. The study was approved by the Research Ethics Committee of Henan Normal University.

Before MC-LR-exposure, the logarithm growth cells were trypsinized by 0.25% of trypsin, collected, and then inoculated in a 6-well culture plate (1.5 × 10^4^ cells per well). After 24 h of culture, the original medium was discarded and the new medium with MC-LR-solution at concentrations of 0 (1.9 mL of RPMI-1640 + 0.1 mL PBS), 10 (1.9 mL of RPMI-1640 + 0.1 mL of 200 μM MC-LR-solution), and 50 μM (1.9 mL of RPMI-1640 + 0.1 mL of 1 000 μM MC-LR-solution) was added to the culture plate (each concentration included two wells) and the cells were cultured for another 24 h. The test was repeated once (*n* = 2). The concentrations of MC-LR used for the toxicity test were obtained according to the results of the MTT cell viability assay conducted in our preliminary experiment (Figure S5).

### 4.3. RNA Extraction

After 24 h of MC-LR-exposure, the cells were collected as described above and washed twice with PBS to remove MC-LR, and then the total RNA was isolated from the cells using an RNAiso Plus kit (Takara, Dalian, China) according to manufacturer’s instructions. The quality analysis of RNA is shown in Figure S6.

### 4.4. High-Throughput Sequencing

Total RNA concentration and purity were determined spectrophotometrically according to the method of Sambrook and Russel [61]. Totally, 30 μg of RNA from 3 independent-treatment groups was mixed together as one sample and then subjected to BGI (Huada Genomics Institute Co. Ltd., Shenzhen, China) for deep sequencing.

### 4.5. Bioinformatic Analysis of Small RNA Sequences

After deep sequencing, the sequence reads went through the data cleaning procedure as follow: (1) filter and remove the low quality reads; (2) remove reads with 5′ primer contaminants; (3) remove reads without 3′ primer; (4) remove reads without the insert tag; (5) remove reads with poly A; (6) remove reads shorter than 18 nt; and (7) summarize the length distribution of the clean reads and retain only trimmed reads of sizes from 18 to 30 nt. Then, overview of small RNA library was obtained, including length distribution, common and specific sequences between samples, genome mapping, and annotation. Filtered sequences were mapped to human genome (https://www.ncbi.nlm.nih.gov/genome/?term=Homo_sapiens) using SOAP algorithm [62]. The clean tags that could not be annotated to any category were taken to predict novel miRNAs using Mirdeep.

### 4.6. Analysis of Differentially Expressed miRNAs

The differential expressed miRNAs between the MC-LR-treated cells and control cells were compared using ExpDiff method as described by Huang et al. [63]. Firstly, normalize the expression of miRNA in control and MC-LR-treatment samples to get the expression of transcript per million and then calculate fold-change and *p*-value from the normalized expression and generate the log2 ratio plot and scatter plot.

### 4.7. Target Prediction of Differentially Expressed miRNAs and GO and KEGG Pathway Analysis

The targetscan and miRanda algorithm were used to identify the target genes of differentially expressed miRNAs as described by Enright et al. [64] using human target reference genes (ftp://ftp.ncbi.nlm.nih.gov/genomes/Homo_sapiens/RNA/). The GO functional analysis of the putative target gene was performed by GO program (www.geneontology.org) as described by Boyle et al. [65] and Ye et al. [66] and the KEGG pathway (http://www.genome.jp/kegg/pathway. html) analysis was performed as described by Kanehisa et al. [67].

### 4.8. qPCR Verification of Differentially Expressed miRNAs

Isolation of miRNAs, synthesis of first-strand cDNA, and qPCR were performed following the method described previously [42]. The miRNAs were isolated from the cells using miRNA Purification Kit (CoWin Biosciences, Beijing, China). The purified miRNAs were then used for poly (A)-adding and the first-strand cDNA synthesized using miRNA cDNA Kit (CoWin Biosciences, Beijing, China). The expressions of miRNAs in HepG2 cells were determined by qPCR using the miRNA Real-Time PCR Assay Kit (CoWin Biosciences, Beijing, China) according to manufacturer’s instructions. The upstream primers and miRNA target specific primers were designed based on the sequences retrievable from miRbase as shown in Table 2. The downstream primers were obtained from the miRNA Real-Time PCR Assay Kit (CoWin Biosciences, Beijing, China). The amount of target miRNA was normalized to U6 mRNA (an internal control) and was determined using the formula 2^−∆∆Ct^ [68].

### 4.9. qPCR Verification the Targets miRNAs

Before MC-LR-exposure, the logarithm growth cells were trypsinized by 0.25% of trypsin, collected, and then inoculated in four 6-well culture plates (1.5 × 10^4^ cells per well) for 24 h and then the medium was discarded and replaced with the fresh complete RPMI-1640 medium containing various concentrations of MC-LR (0, 0.1, 0.5, 1, 5, and 10 μM) for another 3, 6, 12, and 24 h. After MC-LR exposure, the cells were collected from one 6-well culture plate and the total RNA isolation, synthesis of first-strand cDNA, and qPCR were as described above, respectively. The experiment was performed in triplicate, and in total 12 plates was used.

### 4.10. Statistical Analysis

The target miRNA expression data were analyzed using a one-way analysis of variance followed by least significant difference determination with SPSS 13.0 (SPSS, Chicago, IL, USA) for Windows. A *p* value less than 0.05 were considered to be statistically significant (* < 0.05 and ** < 0.01).

## Figures and Tables

**Table 1:** The species and quantity of miRNAs with significantly different expressions in HepG2 cells between the control and 10 and 50 μM MC-LR treatment groups.

miR_name	0-std	10-std	50-std	10 μM-vs-0 Fold-Change	50 μM-vs-0 Fold-Change	*p*-Value
has-miR-1180-3p	5.6930	2.8285	-	−1.0092	-	**
hsa-miR-1180-5p	3.7954	0.9428	-	−2.009	-	**
hsa-miR-149-3p	5.0605	10.8949	23.5600	1.1063	2.2189	**
hsa-miR-217	2.0031	0.8381	-	−1.2570	-	*
hsa-miR-3064-5p	1.3705	0.3143	-	−2.1245	-	*
hsa-miR-324-5p	11.3861	5.2379	-	−1.1202	-	**
hsa-miR-4286	3.9008	1.4666	1.6205	−1.4113	−1.2673	**
hsa-miR-4421	1.7923	0.6285	-	−1.5118	-	*
hsa-miR-4454	1.3705	0.3143	-	−2.1245	-	*
hsa-miR-4485-3p	1.3705	0.4190	-	−1.7097	-	*
hsa-miR-449c-5p	3.3737	6.9140	7.6040	1.0352	1.1724	**
hsa-miR-454-3p	1.0543	2.3047	2.4931	1.1283	1.2417	*
hsa-miR-455-3p	3.6899	1.7809	-	−1.0509	-	*
hsa-miR-499a-5p	4.6388	2.3047	-	−1.0092	-	**
hsa-miR-500a-3p	7.2744	3.5618	3.4904	−1.0302	−1.0594	**
hsa-miR-500a-5p	3.7954	0.6285	0.7479	−2.5943	−2.3433	**
hsa-miR-500b-5p	3.7954	0.6285	0.7479	−2.5943	−2.3433	**
hsa-miR-548v	0.5271	1.4666	-	1.4763	-	*
hsa-miR-590-5p	2.5302	1.2571	-	−1.0092	-	*
hsa-miR-660-3p	0.2109	1.0476	-	2.3125	-	*
hsa-miR-6858-5p	1.0543	0.01	-	−6.7201	-	**
hsa-let-7b-3p	2.3194	-	0.9973	-	−1.2177	*
hsa-miR-106a-5p	0.2109	-	1.4959	-	2.8264	**
hsa-miR-1247-3p	2.6357	-	0.7479	-	−1.8173	**
hsa-miR-1247-5p	5.5876	-	2.6178	-	−1.0939	**
hsa-miR-1256	1.7923	-	0.4986	-	−1.8459	*
hsa-miR-1276	0.3163	-	1.1219	-	1.8266	*
hsa-miR-184	0.5271	-	4.2383	-	3.0073	**
hsa-miR-192-5p	244.9062	-	502.3647	-	1.0365	**
hsa-miR-194-5p	2.9519	-	9.9725	-	1.7563	**
hsa-miR-2116-3p	1.3705	-	0.3740	-	−1.8736	*
hsa-miR-21-3p	14.6543	-	30.1668	-	1.0416	**
hsa-miR-27a-5p	2.5302	-	7.6040	-	1.5875	**
hsa-miR-29a-5p	0.5271	-	1.4959	-	1.5049	*
hsa-miR-3127-5p	1.3705	-	4.2383	-	1.6288	**
hsa-miR-3162-5p	1.2651	-	2.7424	-	1.1162	*
hsa-miR-3614-5p	0.2109	-	1.1219	-	2.4113	*
hsa-miR-3615	0.9488	-	2.1192	-	1.1593	*
hsa-miR-362-3p	2.9519	-	1.3712	-	−1.1062	*
hsa-miR-3916	1.5814	-	0.3740	-	−2.0801	*
hsa-miR-4440	1.0543	-	0.01	-	−6.7201	**
hsa-miR-4470	1.0543	-	0.2493	-	−2.0803	*
hsa-miR-4710	1.8977	-	0.4986	-	−1.9283	**
hsa-miR-4739	4.1116	-	1.8698	-	−1.1368	**
hsa-miR-4741	2.0031	-	4.7369	-	1.2417	**
hsa-miR-6515-5p	0.3163	-	1.2466	-	1.9786	*
hsa-miR-6758-5p	1.0543	-	0.2493	-	−2.0803	*
hsa-miR-6805-5p	1.8977	-	4.2383	-	1.1592	**
hsa-miR-6821-5p	0.2109	-	1.1219	-	2.4113	*
hsa-miR-766-3p	2.5302	-	0.6233	-	−2.0213	**
hsa-miR-9-5p	0.01	-	1.6205	-	7.3403	**

Note: * < 0.05 and ** < 0.01.

**Table 2:** Specific primers used for the qPCR in this study.

miRNAs	Accession Number	Primers [5′→3′]
hsa-miR-1180-3p	MIMAT0005825	GCTCGCGTGGGTGTGTAAAAA
hsa-miR-1180-5p	MIMAT0026735	CTGCTGGACCCACCCGAAAA
hsa-miR-1247-3p	MIMAT0022721	ACCCCGGGAACGTCGAGAAA
hsa-miR-149-3p	MIMAT0004609	AGGAGGGAGGGAGGGACAAA
hsa-miR-192-5p	MIMAT0000222	GGGCTCTGACCTATGAATTGAA
hsa-miR-194-5p	MIMAT0000460	CGCGTAACAGCAACTCCAAAAA
hsa-miR-21-3p	MIMAT0004494	GGCAACACCAGTCGATGAAAAA
hsa-miR-27a-5p	MIMAT0004501	CAGGGCTTAGCTGCTTGTGAA
hsa-miR-29a-5p	MIMAT0004503	GACTGATTTCTTTTGGTGTTCAG
hsa-miR-324-5p	MIMAT0000761	CCCCTAGGGCATTGGTGTAAA
hsa-miR-4286	MI0015894	ACCCCACTCCTGGTACCAAAA
hsa-miR-449c-5p	MIMAT0010251	CAGTGTATTGCTAGCGGCTGT
hsa-miR-454-3p	MIMAT0003885	GGTGCAATATTGCTTATAGGGA
hsa-miR-4710	MI0017344	GTGGGGTGAGGGCAGGTAAA
hsa-miR-499a-5p	MIMAT0002870	CGGCTGTTAAGACTTGCAGAAA
hsa-miR-500a-3p	MIMAT0002871	GCAATGCACCTGGGCAAGAAA
hsa-miR-500a-5p	MIMAT0004773	ATCCTTGCTACCTGGGTGAGA
hsa-miR-500b-5p	MIMAT0016925	AATCCTTGCTACCTGGGTAAAA
hsa-miR-548v	MIMAT0015020	CAGTTACTTTTGCACCAGCCTA
hsa-miR-660-3p	MIMAT0022711	CTGTGTGCATGGATTACAGGAA
hsa-miR-9-5p	MIMAT0000441	GGCTCTTTGGTTATCTAGCTGA
U6		GCTTCGGCAGCACATATACTAA
		GCTTCACGAATTTGCGTGTCAT

## Supplementary Materials

The following are available online at www.mdpi.com/2072-6651/9/1/23/s1, Figure S1: Length distributions of small RNA reads in HepG2 cells from the control and MC-LR treatment groups, Figure S2: First nucleotide bias of 18–30 nt miRNA tags, Figure S3: Small RNA annotation for the control and MC-LR treatment groups, Figure S4: The structure of novel miRNA candidates, Figure S5: Viability of HepG2 cells exposed to various concentrations of MC-LR, Figure S6: Quality determination of the tolal RNA from the HepG2 cells, Table S1: Summary of small RNA sequencing data, Table S2: Summary of known miRNAs in control and MC-LR treatment groups, Table S3: Statistics of predicted target genes for differentially expressed miRNAs, Table S4: Significantly enriched biological processes for the candidate target genes of differentially expressed miRNAs in HepG2 cells between the control and 10 μM group by using GO enrichment analysis, Table S5: Significantly enriched biological processes for the candidate target genes of differentially expressed miRNAs in HepG2 cells between the control and 50 μM group by using GO enrichment analysis, Table S6: Enriched 20 KEGG pathways for the candidate target genes of differentially expressed miRNAs in HepG2 cells between the control and 10 μM group, Table S7: Enriched 20 KEGG pathways for the candidate target genes of differentially expressed miRNAs in HepG2 cells between the control and 50 μM group, Table S8: Statistics of the quantity of novel miRNAs in HepG2 cells between the control and MC-LR treatment groups.
