# Supplementary material for: Analysis of MicroRNA Expression Profiling Involved in MC-LR-Induced Cytotoxicity by High-Throughput Sequencing
Source: Toxins (Basel). 2017 Jan 7;9(1):23. doi: 10.3390/toxins9010023 (PMC5308255; doi:10.3390/toxins9010023)
Supplement: Supplementary file 1 [file toxins-09-00023-s001.pdf]

Supplementary Materials: Analysis of MicroRNA Expression Profiling Involved in MC-LR-Induced Cytotoxicity by High-Throughput Sequencing

Junguo Ma, Yuanyuan Li, Lan Yao and Xiaoyu Li

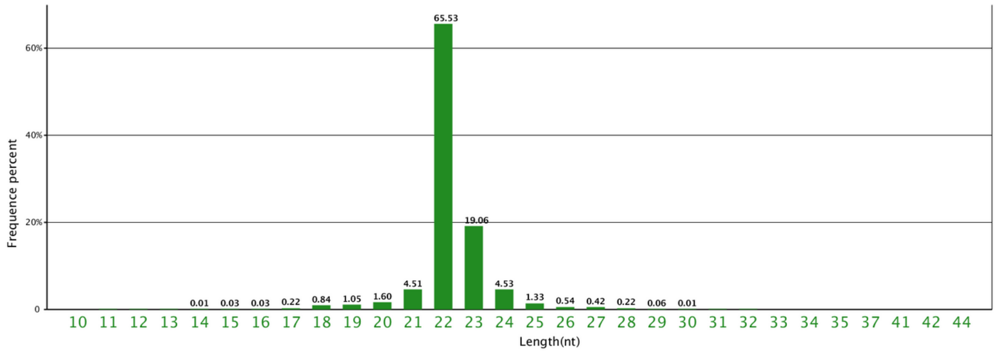

(**A**)

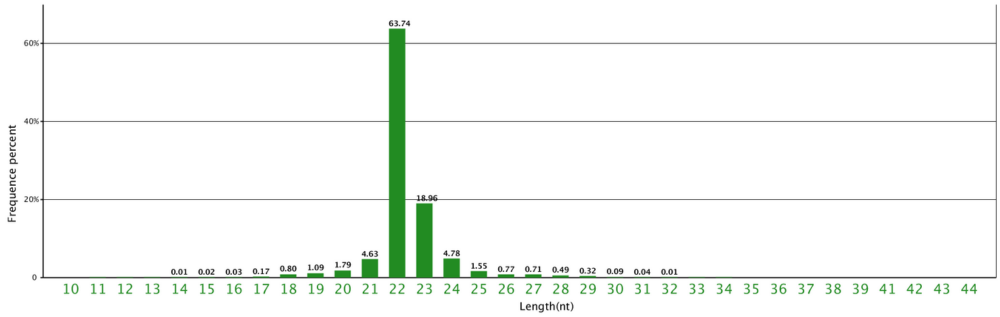

(**B**)

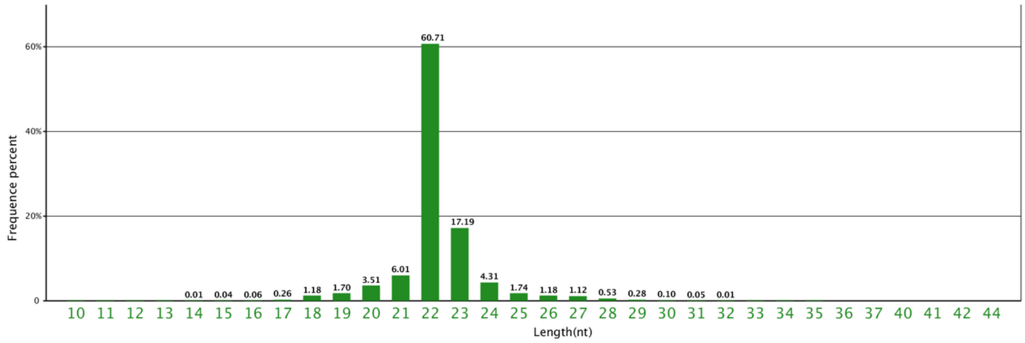

(**C**)

Figure S1. Length distributions of small RNA reads in HepG2 cells from the control and MC-LR treatment groups. (**A**) Control group; (**B**) 10 μM; (**C**) 50 μM.

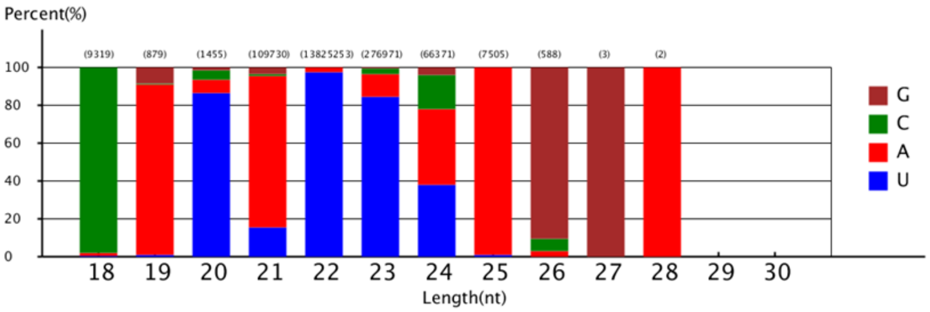

(**A**)

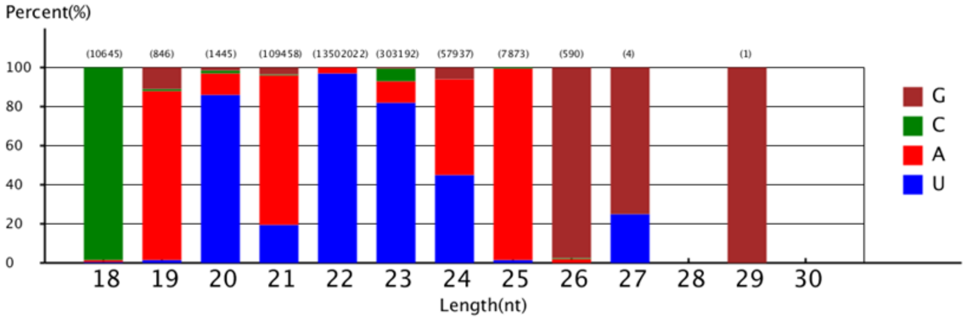

(**B**)

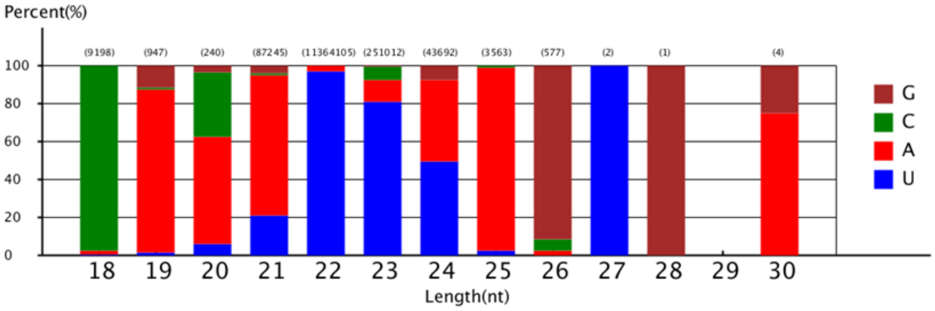

(**C**)

Figure S2. First nucleotide bias of 18–30 nt miRNA tags. (**A**) control; (**B**) 10 μM; (**C**) 50 μM.

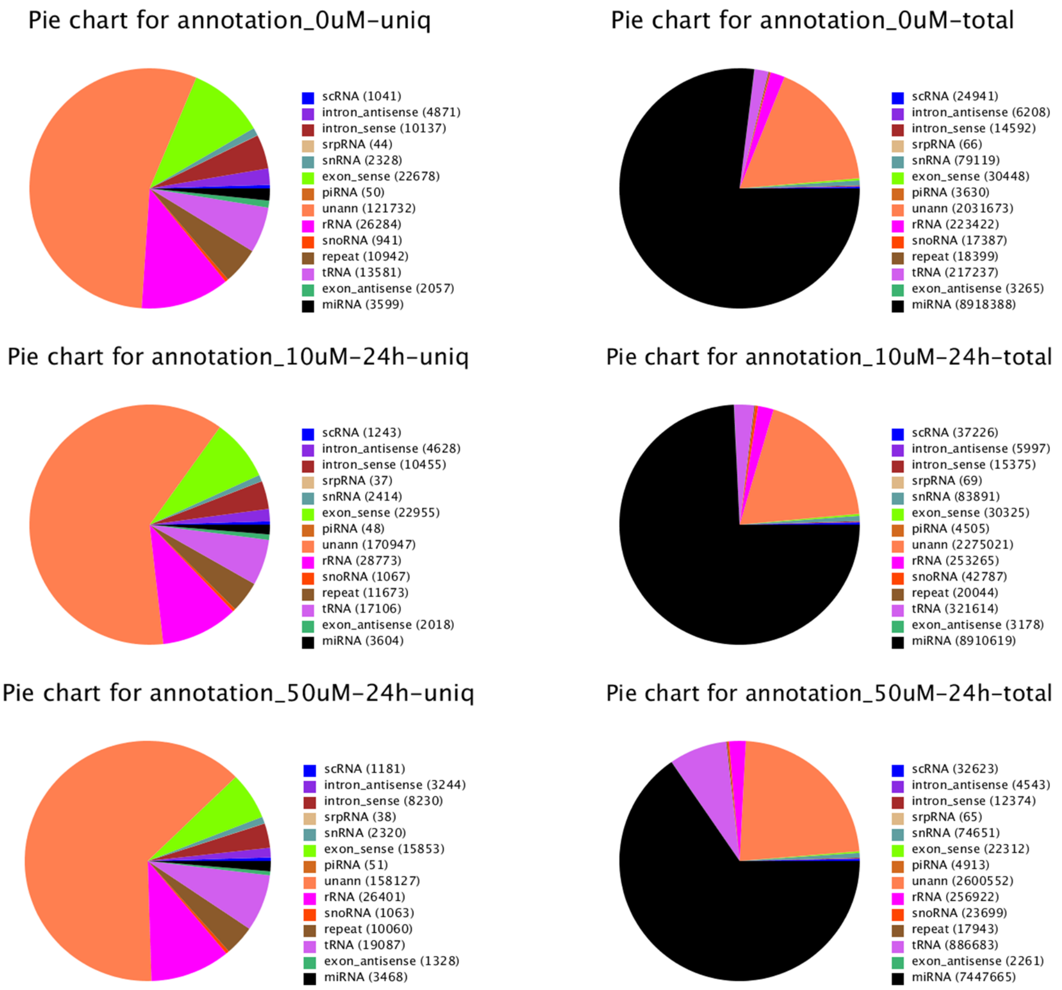

Figure S3. Small RNA annotation for the control and MC-LR treatment groups.

> novel-mir-39

TCGGGCGGGAGTGGTGGCTTTT

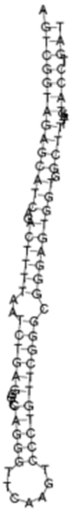

> novel-mir-14

GAGTTAGCGGGGAGTGATATATT

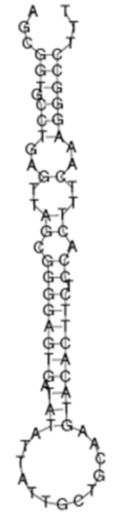

Figure S4. The structure of novel miRNA candidates.

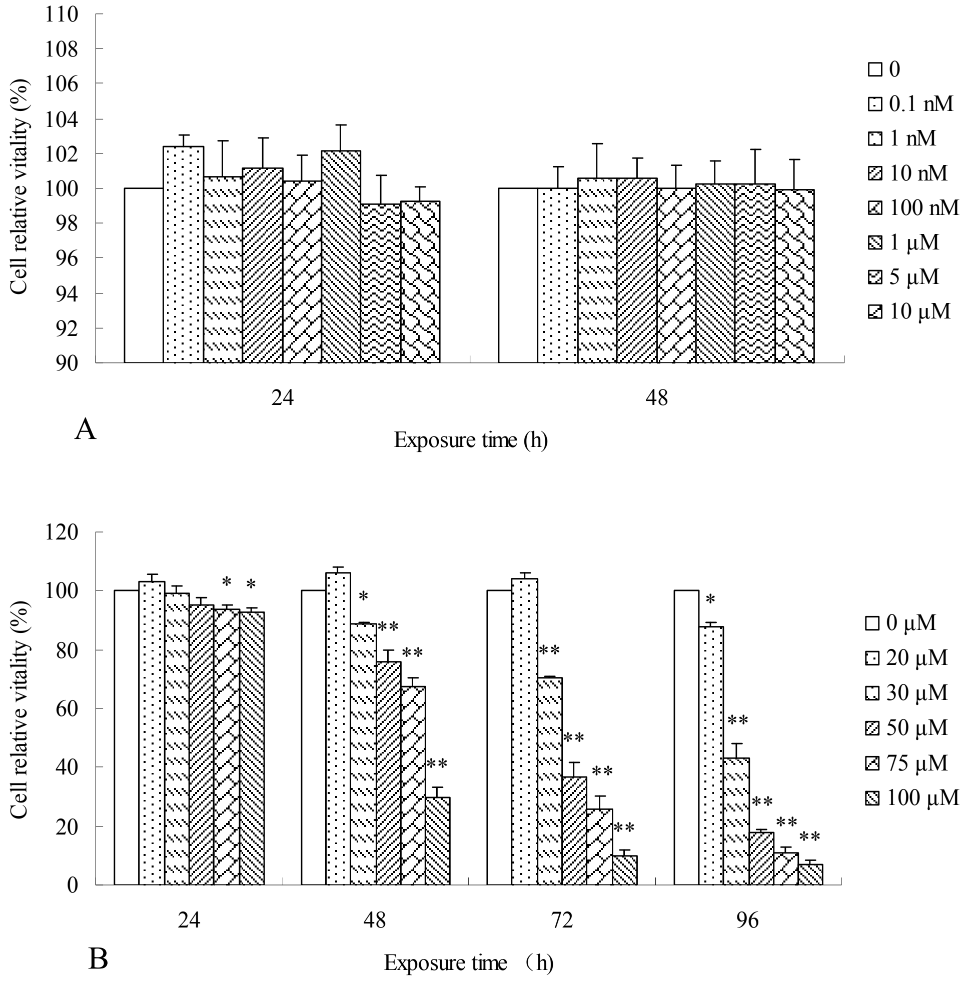

Figure S5. Viability of HepG2 cells exposed to various concentrations of MC-LR. (**A**) 0.1 nM–10 μM MC-LR exposure for 48 h; (**B**) 20–100 μM MC-LR exposure for 96 h.

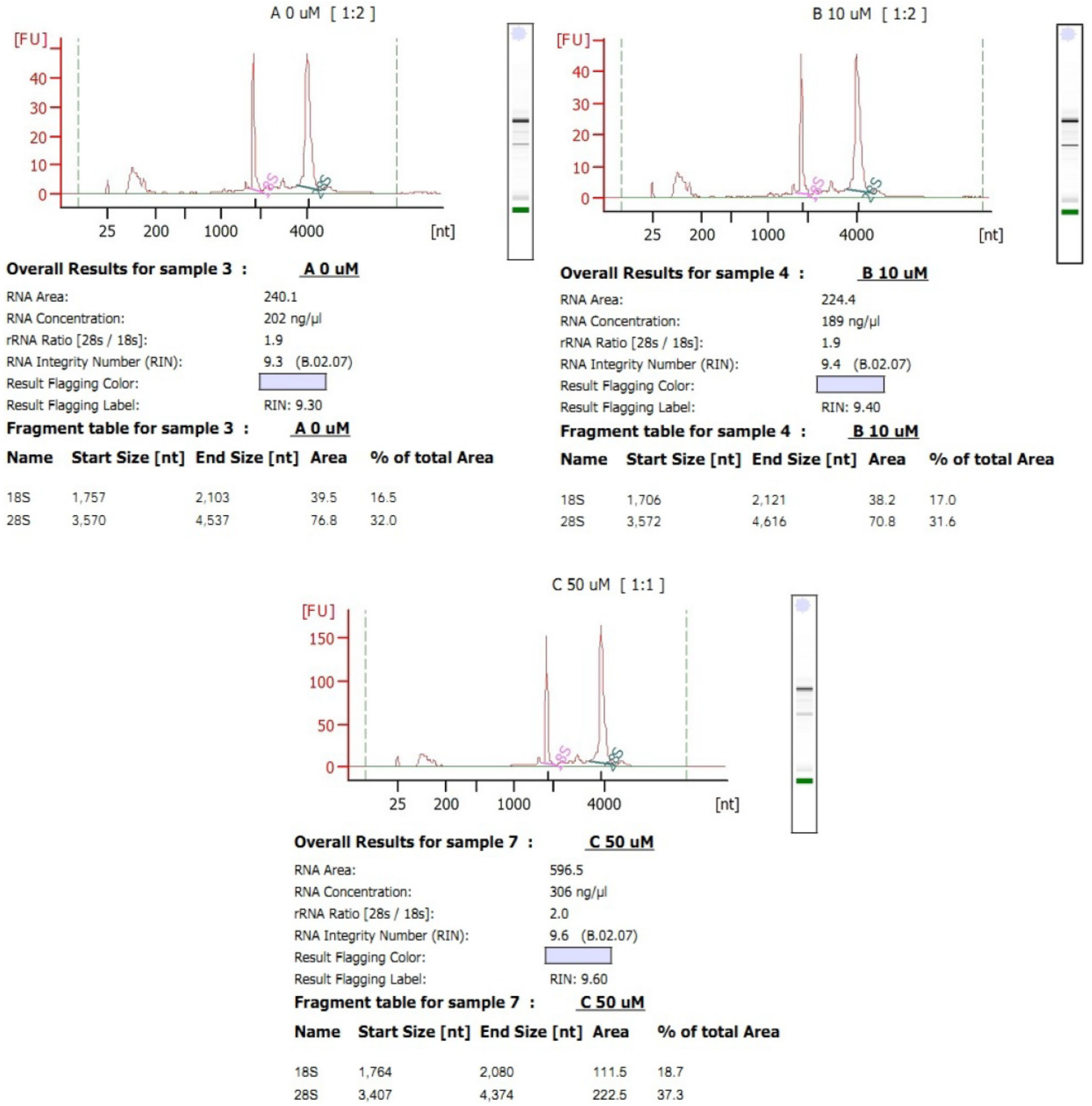

Figure S6. Quality determination of the tolal RNA from the HepG2 cells. (**A**) Control group; (**B**) 10 µM; (**C**) 50 µM.

Table S1. Summary of small RNA sequencing data.

Reads Types	Control		10 µM MC-LR-Treated Cells		50 µM MC-LR-Treated Cells	
	Reads Number	Percentage	Read Number	Percentage	Read Number	Percentage
Total reads	11,883,540		12,304,052		11,856,736	
High quality	11,816,395	100%	12,233,110	100%	11,801,079	100%
3′ adapter null	4875	0.04%	5486	0.04%	3339	0.03%
Insert null	8518	0.07%	10,821	0.09%	14,986	0.13%
5′ adapter contaminants	178,020	1.51%	182,917	1.50%	349,717	2.96%
Smaller than 18 nt	35,265	0.30%	28,849	0.24%	44,825	0.38%
Poly A	942	0.01%	1121	0.01%	1006	0.01%
Clean reads	11,588,775	98.07%	12,003,916	98.13%	11,387,206	96.49%

Table S2. Summary of known miRNAs in control and MC-LR treatment groups.

Category	miRNA	miRNA-5p	miRNA-3p	miRNA Precursors
Known miRNAs in miRbase	821	879	888	1881
Control	192	392	267	725
10 μM	189	378	248	716
50 μM	193	378	262	725

Table S3. Statistics of predicted target genes for differentially expressed miRNAs.

Category	0 μM vs. 10 μM			0 μM vs. 50 μM		
	Differentially Expressed miRNAs	Target Gene Number	Target Site Number	miRNA	Targets Gene Number	Target Site Number
targetscan	21	39078	387962	37	39902	715147
miRanda	21	37652	231702	37	39267	430018
Result	21	37566	-	37	39174	-

Table S4. Significantly enriched biological processes for the candidate target genes of differentially expressed miRNAs in HepG2 cells between the control and 10 μM group by using GO enrichment analysis.

Gene Ontology Terms	Cluster Frequency	Genome Frequency of Use	Corrected *p*-Value
Cell periphery	2938 out of 29,499 genes, 10.0%	2998 out of 30,910 genes, 9.7%	1.60×10^{-12}
Plasma membrane	2905 out of 29,499 genes, 9.8%	2965 out of 30,910 genes, 9.6%	4.25×10^{-12}
Plasma membrane part	2760 out of 29,499 genes, 9.4%	2818 out of 30,910 genes, 9.1%	5.13×10^{-11}
Cell projection	1859 out of 29,499 genes, 6.3%	1894 out of 30,910 genes, 6.1%	2.01×10^{-8}
Cytoskeleton	2582 out of 29,499 genes, 8.8%	2645 out of 30,910 genes, 8.6%	3.08×10^{-7}
Cytoskeletal part	1475 out of 29,499 genes, 5.0%	1503 out of 30,910 genes, 4.9%	2.87×10^{-6}
Integral to membrane	2920 out of 29,499 genes, 9.9%	3001 out of 30,910 genes, 9.7%	1.04×10^{-5}
Neuron projection	985 out of 29,499 genes, 3.3%	1001 out of 30,910 genes, 3.2%	7.15×10^{-5}
Cell junction	817 out of 29,499 genes, 2.8%	829 out of 30,910 genes, 2.7%	0.00020
Intrinsic to plasma membrane	470 out of 29,499 genes, 1.6%	473 out of 30,910 genes, 1.5%	0.00020
Cell leading edge	509 out of 29,499 genes, 1.7%	513 out of 30,910 genes, 1.7%	0.00025
Integral to plasma membrane	457 out of 29,499 genes, 1.5%	460 out of 30,910 genes, 1.5%	0.00035
Microtubule cytoskeleton	1395 out of 29,499 genes, 4.7%	1428 out of 30,910 genes, 4.6%	0.00157
Cell	28,667 out of 29,499 genes, 97.2%	30,007 out of 30,910 genes, 97.1%	0.00285
Cell part	28,667 out of 29,499 genes, 97.2%	30,007 out of 30,910 genes, 97.1%	0.00285
Intrinsic to organelle membrane	311 out of 29,499 genes, 1.1%	312 out of 30,910 genes, 1.0%	0.00297
Microtubule organizing center	627 out of 29,499 genes, 2.1%	636 out of 30,910 genes, 2.1%	0.00371
Golgi membrane	304 out of 29,499 genes, 1.0%	305 out of 30,910 genes, 1.0%	0.00405
Anchoring junction	443 out of 29,499 genes, 1.5%	448 out of 30,910 genes, 1.4%	0.01502
Insoluble fraction	2092 out of 29,499 genes, 7.1%	2155 out of 30,910 genes, 7.0%	0.01688
Membrane	13,293 out of 29,499 genes, 45.1%	13,855 out of 30,910 genes, 44.8%	0.02543
Basolateral plasma membrane	390 out of 29,499 genes, 1.3%	394 out of 30,910 genes, 1.3%	0.02605
Coated vesicle	387 out of 29,499 genes, 1.3%	391 out of 30,910 genes, 1.3%	0.02916

Table S5. Significantly enriched biological processes for the candidate target genes of differentially expressed miRNAs in HepG2 cells between the control and 50 μM group by using GO enrichment analysis.

Gene Ontology Terms	Cluster Frequency	Genome Frequency of Use	Corrected *p*-Value
Integral to membrane	2992 out of 30,554 genes, 9.8%	3001 out of 30,910 genes, 9.7%	3.51×10^{-5}
Cell periphery	2983 out of 30,554 genes, 9.8%	2998 out of 30,910 genes, 9.7%	0.03409
Plasma membrane	2950 out of 30,554 genes, 9.7%	2965 out of 30,910 genes, 9.6%	0.04362

Table S6. Enriched 20 KEGG pathways for the candidate target genes of differentially expressed miRNAs in HepG2 cells between the control and 10 μM group.

Sequence Number	Pathway	Target Genes with Pathway Annotation (30,782)	All Genes of the Species with Pathway Annotation (32,154)	*Q* Value	Pathway ID
1	Transcriptional misregulation in cancer	2777 (9.02%)	2851 (8.87%)	0.0001604321	ko05202
2	Dilated cardiomyopathy	699 (2.27%)	707 (2.2%)	0.0001604321	ko05414
3	Hypertrophic cardiomyopathy (HCM)	680 (2.21%)	688 (2.14%)	0.0002012056	ko05410
4	MAPK signaling pathway	991 (3.22%)	1010 (3.14%)	0.0011832452	ko04010
5	Endocytosis	857 (2.78%)	872 (2.71%)	0.0011832452	ko04144
6	Herpes simplex infection	563 (1.83%)	570 (1.77%)	0.0012157766	ko05168
7	Arrhythmogenic right ventricular cardiomyopathy (ARVC)	397 (1.29%)	400 (1.24%)	0.0012157766	ko05412
8	Tuberculosis	645 (2.1%)	655 (2.04%)	0.0023238166	ko05152
9	Purine metabolism	2554 (8.3%)	2634 (8.19%)	0.0140970537	ko00230
10	Cocaine addiction	229 (0.74%)	230 (0.72%)	0.0149215617	ko05030
11	Biosynthesis of secondary metabolites	2845 (9.24%)	2938 (9.14%)	0.0210462281	ko01110
12	Viral myocarditis	586 (1.9%)	597 (1.86%)	0.0210462281	ko05416
13	Antigen processing and presentation	207 (0.67%)	208 (0.65%)	0.0275114985	ko04612
14	Pyrimidine metabolism	2298 (7.47%)	2373 (7.38%)	0.0516396347	ko00240
15	Influenza A	605 (1.97%)	618 (1.92%)	0.0516396347	ko05164
16	Glycolysis/Gluconeogenesis	185 (0.6%)	186 (0.58%)	0.0530529544	ko00010
17	Cholinergic synapse	330 (1.07%)	335 (1.04%)	0.0687764858	ko04725
18	Endocrine and other factor-regulated calcium reabsorption	820 (2.66%)	841 (2.62%)	0.0687764858	ko04961
19	Phagosome	643 (2.09%)	658 (2.05%)	0.0687764858	ko04145
20	Bacterial invasion of epithelial cells	391 (1.27%)	398 (1.24%)	0.0713390445	ko05100

Table S7. Enriched 20 KEGG pathways for the candidate target genes of differentially expressed miRNAs in HepG2 cells between the control and 50 μM group.

Sequence Number	Pathway	Target Genes with Pathway Annotation (31,785)	All Genes of the Species with Pathway Annotation (32,154)	*Q* Value	Pathway ID
1	Purine metabolism	2624 (8.26%)	2634 (8.19%)	0.002971387	ko00230
2	Transcriptional misregulation in cancer	2837 (8.93%)	2851 (8.87%)	0.016436107	ko05202
3	Pyrimidine metabolism	2362 (7.43%)	2373 (7.38%)	0.024226428	ko00240
4	MAPK signaling pathway	1007 (3.17%)	1010 (3.14%)	0.210478995	ko04010
5	Focal adhesion	978 (3.08%)	981 (3.05%)	0.214672559	ko04510
6	Pancreatic secretion	945 (2.97%)	948 (2.95%)	0.214672559	ko04972
7	Biosynthesis of secondary metabolites	2918 (9.18%)	2938 (9.14%)	0.214672559	ko01110
8	Amoebiasis	447 (1.41%)	447 (1.39%)	0.214672559	ko05146
9	Axon guidance	601 (1.89%)	602 (1.87%)	0.251439209	ko04360
10	Cardiac muscle contraction	1132 (3.56%)	1137 (3.54%)	0.270541735	ko04260
11	Arrhythmogenic right ventricular cardiomyopathy (ARVC)	400 (1.26%)	400 (1.24%)	0.270541735	ko05412
12	Dilated cardiomyopathy	705 (2.22%)	707 (2.2%)	0.299621458	ko05414
13	Hypertrophic cardiomyopathy (HCM)	686 (2.16%)	688 (2.14%)	0.325081682	ko05410
14	Aldosterone-regulated sodium reabsorption	804 (2.53%)	807 (2.51%)	0.325081682	ko04960
15	Wnt signaling pathway	518 (1.63%)	519 (1.61%)	0.325081682	ko04310
16	Carbohydrate digestion and absorption	794 (2.5%)	797 (2.48%)	0.325081682	ko04973
17	NF-kappa B signaling pathway	347 (1.09%)	347 (1.08%)	0.325081682	ko04064
18	Bile secretion	881 (2.77%)	885 (2.75%)	0.420171933	ko04976
19	Proximal tubule bicarbonate reclamation	724 (2.28%)	727 (2.26%)	0.488366532	ko04964
20	Epstein-Barr virus infection	591 (1.86%)	593 (1.84%)	0.488366532	ko05169

Table S8. Statistics of the quantity of novel miRNAs in HepG2 cells between the control and MC-LR treatment groups.

miRNA Name	0 μM	10 μM	50 μM
novel-mir-39	18,132	18,507	18,365
novel-mir-14	311	431	374
novel-mir-33	130	144	142
novel-mir-12	98	58	66
novel-mir-4	87	83	70
novel-mir-23	72	71	79
novel-mir-6	70	74	58
novel-mir-2	30	22	16
novel-mir-46	20	26	19
novel-mir-48	17	22	17
novel-mir-11	17	14	10
novel-mir-38	15	16	15
novel-mir-3	14	17	8
novel-mir-18	12	18	9
novel-mir-44	12	9	14
novel-mir-45	11	12	18
novel-mir-21	11	6	11
novel-mir-43	7	9	8
